# Racial and Ethnic Disparities Within Social Determinants of Health Amongst Patients With Systemic Lupus Erythematosus

**DOI:** 10.7759/cureus.64453

**Published:** 2024-07-13

**Authors:** Sunaina Addanki, Krina Patel, Kriya Shah, Lisa Patel, McHenry Mauger, Anita Laloo, Vijay Rajput

**Affiliations:** 1 Medicine, Nova Southeastern University Dr. Kiran C. Patel College of Allopathic Medicine, Fort Lauderdale, USA; 2 Osteopathic Medicine, Nova Southeastern University Dr. Kiran C. Patel College of Osteopathic Medicine, Fort Lauderdale, USA; 3 Biostatistics, Nova Southeastern University Dr. Kiran C. Patel College of Allopathic Medicine, Fort Lauderdale, USA; 4 Rheumatology, Nova Southeastern University Dr. Kiran C. Patel College of Osteopathic Medicine, Fort Lauderdale, USA; 5 Medical Education, Nova Southeastern University Dr. Kiran C. Patel College of Allopathic Medicine, Fort Lauderdale, USA

**Keywords:** lupus, ethnicity, race, social determinants of health (sdoh), systemic lupus erythematous (sle)

## Abstract

Introduction

This study aims to identify the influence of social determinants of health (SDoH) on patients with systemic lupus erythematosus (SLE), emphasizing racial and ethnic disparities in healthcare.

Methods

A cross-sectional study used the National Institute of Health’s (NIH) All of Us Research Program (AoU). From 727,000 patients, SLE patients were categorized by race, ethnicity, and responses to the Social Determinants of Health survey from May 2018 until March 2023. Survey questions addressed transportation access, neighborhood safety, provider biases, and food insecurity. JMP Pro 16.0 and R 4.2.2 were used for statistical analysis.

Results

Significant racial disparities were evident amongst SLE patients for transportation access, neighborhood safety, food security, and respect from healthcare providers (p-value < 0.001). African Americans, Asians, and White participants showed different perceptions regarding neighborhood crime, healthcare provider courtesy, and feeling unheard by providers, with respective p-values of 0.001, 0.010, and 0.023. Hispanic participants perceived higher neighborhood crime rates, felt unsafe during nighttime walks, felt unheard by healthcare providers, and reported worrying about food security compared to non-Hispanic participants, with respective p-values of 0.003, 0.003, 0.009, and <0.001.

Discussion

SLE is affected by access to care, treatments, stress, and lifestyle habits. Therefore, identifying SDoH for SLE patients is critical as it impacts disease progression, leading to delays in diagnosis, improper management, and worsening morbidity.

Conclusion

Targeted social and community-based interventions may improve access to care, identify implicit biases among providers, and alleviate food insecurity.

## Introduction

Systemic lupus erythematosus (SLE) is an autoimmune disease that can present with a variety of indolent symptoms and complications. There are no clear etiological factors attributed to this chronic, progressive disease. The pathophysiology of SLE is multifactorial, with several proposed mechanisms hypothesized to play a role in the development and progression of the disease. The symptoms and severity of SLE vary from person to person. The diagnosis is based on the European Alliance of Associations for Rheumatology/American College of Rheumatology (EULAR/ACR) criteria, where different symptoms are weighted and scored. The most prevalent symptoms are fever, malaise, and the distinctive malar rash that spares the nasolabial folds. Other organ systems are often involved, with symptoms ranging from arthritis, anemia, seizures, depression, and pleuritis, with the most severe presentation being lupus nephritis [1]. Follow-up doctor visits are determined based on the severity of the disease. For stable, mild disease, follow-up is recommended every six months. However, for patients with more severe symptoms with cardiac or renal involvement, follow-up is encouraged every month to three months [2]. While rheumatological symptoms influence the course of SLE, one of the most prevalent causes of mortality in SLE patients is related to cardiovascular complications [1].

Epidemiology and burden of the disease

Based on multiple population-based registries, the SLE incidence rate is estimated to be 5.1 per 100,000 people [3]. The prevalence of SLE in the United States is estimated to be 72.8 per 100,000. SLE prevalence is nine times higher in women and also has the highest rate in black females (230.9 per 100,000), followed by Hispanic females (120.7) [4]. The California Lupus Surveillance Project followed 812 patients diagnosed with SLE from 2007-2009 for 10 years and found that mortality rates were highest in blacks (25%), followed by Asians (15.3%), and then whites (14.4%) [5].

In a study done by Maningding et al., blacks, Asian/Pacific Islanders, and Hispanics had a higher incidence of renal complications and progression of the disease compared to whites [6]. Many factors influence the outcomes of the disease, such as genetics, hormones, adherence, behaviors, and social determinants of health (SDoH). Recent research has shown that the SDoH may result in significant disparities within diseases [7]. SDoH is defined as non-medical elements influencing health outcomes and quality of life. Examples include income, education, neighborhood environment, access to healthcare, food security, discrimination, mistreatment in healthcare, and social support [8]. When correlating the outcome of perceived general health in those with lower income and educational levels/health literacy, they had more unmet healthcare needs, higher mortality risk, and worse postoperative outcomes [7]. Living in a disadvantaged neighborhood with limited access to healthcare also contributed to lower chances of obtaining preventative care [9]. For example, Chicago areas that had larger decreases in violent crime for 14 years also had a higher decline in cardiovascular mortality rates [10]. Furthermore, food security is associated with improved health outcomes, as those using the Supplemental Nutrition Assistance Program (SNAP) have decreased avoidable hospitalizations and emergency room visits [11]. Racial discrimination and mistreatment in healthcare have been linked to a variety of poorer outcomes, such as lower life satisfaction, including both physical and mental health [12]. The final SDoH that is often overlooked is social support. Social support has a large impact on mortality risk, psychological stress, burden of care, and quality of life [13]. uncommon SDoHs like food security, providers’ biases, and access to care due to crime in the neighborhood are not well assessed in different races and ethnicities. This cross-sectional study aims to identify disparities in healthcare, specifically amongst individuals with SLE across different racial and ethnic groups. By evaluating specific SDoH, targeted strategies can help address health disparities among SLE patients.

## Materials and methods

Baseline data from the National Institutes of Health (NIH) All of US Researcher Program (AoU) was used to evaluate the relationships between race, ethnicity, and social determinants of health amongst patients with SLE. Data was collected from May 6, 2018, until March 26, 2023. Within the AoU Workbench, a cloud-based platform, Systematized Nomenclature of Medicine (SNOMED) codes, electronic health record (EHR) measurements, and Social Determinants of Health Survey responses were used to group and identify data. Results comply with the AoU Data and Statistics Dissemination Policy, which disallows disclosure of groups under 20 patients. Datasets included consented data, while participant privacy was protected through a series of data transformations. This study was conducted in compliance with the All of Us IRB approval, which reviews protocol, informed consent, and other participant-facing materials. The IRB follows the regulations and guidance of the Office for Human Research Protections. Consent for each participant was done via an e-consent evaluation and followed the primary e-consent and HIPAA authorizations. AoU Workbench tools such as Cohort Builder were used for selecting groups of participants, while Dataset Builders were utilized for creating datasets to analyze, and workspaces with Jupyter Notebooks were used to analyze the data. We used JMP Pro 16.0 and R 4.2.2 to perform the analysis while using Word to create figures to display significant information.

A cross-sectional study was conducted with individuals with SLE who were identified and analyzed by race and ethnicity in relation to the social determinants of health survey. Five different groups were identified by selecting and excluding certain criteria. Inclusion criteria included age groups from 18 to 120 years old, systemic lupus erythematosus, and responses to the Social Determinants of Health survey. Exclusion criteria were dependent on racial and ethnic identities for each group. The first three groups included the three different race categories: African American, White, and Asian. These groups were compared in terms of the social determinants of health survey responses using difference of proportions tests. The next two groups were categorized based on ethnicity: Hispanic or non-Hispanic and were also compared by the social determinants of health survey responses, for which a difference of proportions analysis was conducted. The SNOMED codes for systemic lupus erythematosus included systemic lupus erythematosus and systemic lupus erythematosus with organ/system involvement. Responses to identify race were Asian, Black or African American, White, or Skip, while ethnicities were identified as Hispanic or Non-Hispanic. 

The social determinants of health survey included 86 total questions, of which nine were selected for this study to encompass pertinent social determinants of health for patients with systemic lupus erythematosus. These questions were selected based on the Lupus Foundation of America, which prioritizes income, education, neighborhood factors, healthcare access, discrimination, and social support [14]. The nine questions that were selected were related to transportation access, neighborhood safety, healthcare provider bias, and food insecurity, all of which can significantly impact patients with SLE. Specific questions relating to income and education levels were not identified in the survey and therefore were not chosen. In terms of statistical analysis, for each question, a difference of proportions test was performed to compare the race and ethnicity groups. Significance was determined utilizing standard statistical procedures, such as a two-proportion z-test, to identify a difference of proportions analysis using JMP Pro 16.0 software. 

## Results

**Figure 1:**
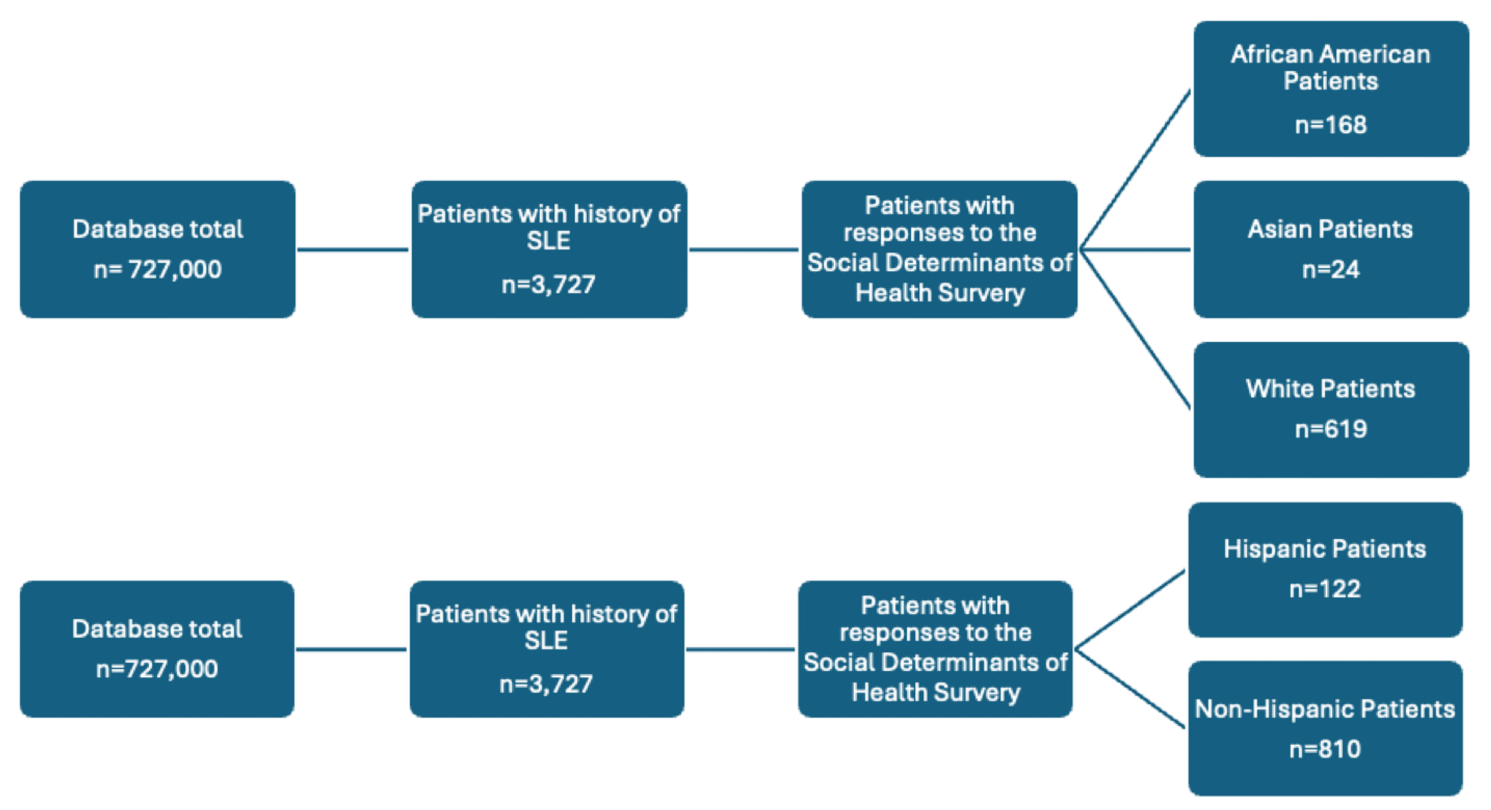
Diagram depicting grouping of patients matched by inclusion and exclusion criteria, including social determinants of health survey, history of SLE, and ethnicity. Ethnicity groups less than 20 were not included in the analysis per the All of Us research program guidelines.

**Figure 2:**
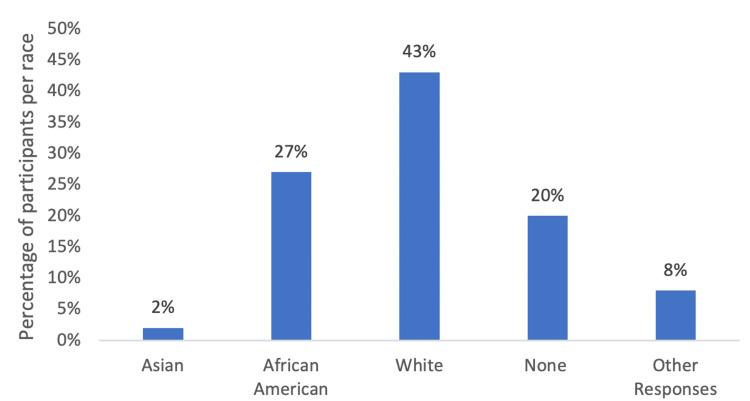
The distribution of races amongst patients with SLE. Other responses indicate “I prefer not to answer,” “none of these,” “skip,” and “more than one population.” The number of participants for these categories was not significant per the All of Us research database guidelines.

**Figure 3:**
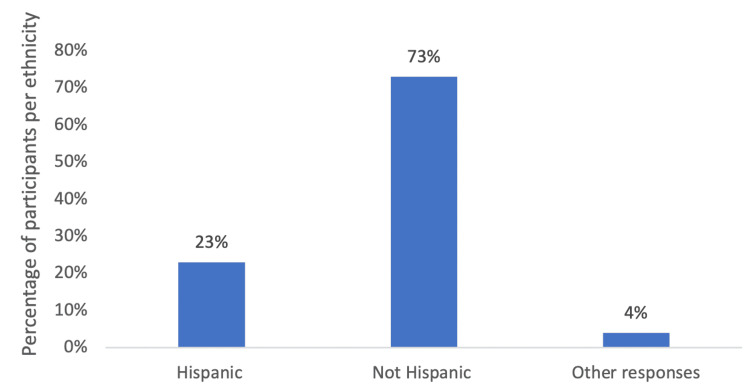
The distribution of ethnicities amongst patients with SLE. Other responses indicated “none of these” and “skip.” The number of participants for these categories was not significant per the All of Us research database guidelines.

Using the All of Us research database, 727,000 participants were matched based on inclusion and exclusion criteria, including race, ethnicity, responses to social determinants of health surveys, and age. Participant grouping based on race yielded three different groups, and grouping based on ethnicity yielded two different groups (Figure 1). In terms of racial analysis for patients with SLE, 43% of the participants were white, 27% of the participants were African American, and 2% of the participants were Asian (Figure 2). In terms of ethnicity, 23% of participants with SLE were Hispanic, while 73% were not Hispanic (Figure 3). 

Racial analysis for the social determinants of health survey responses yielded significant disparities across races. Access to transportation for patients with SLE differed significantly (p<0.001), with African Americans and Asians reporting lower availability compared to white individuals. Perceptions of neighborhood crime (p=0.001) and feelings of safety during nighttime walks within the neighborhood (p<0.001) were significantly different, with African Americans and Asians feeling more unsafe compared to white individuals. Significant disparities were noted amongst the African American responses regarding experiences in healthcare facilities, such as feeling treated with less courtesy (p=0.010), less respect (p=0.023), and not being heard by healthcare providers (0.002). Dissatisfaction with service at healthcare facilities (p=0.001) and worries relating to food and financial security (p<0.001) were significantly higher in African Americans compared to Asian and White individuals (Table 1). These results corresponded to differences in responses such as “most of the time,” “rarely,” “sometimes,” “often true” or “sometimes true” based on the specific question. 

**Table 1:** Social Determinants of Health Survey questions and responses for each race. A difference of proportions analysis with p-values is indicated for each question. Data is presented throughout as 'n(%)' (*) represents significance in relation to p<0.05 Significance was determined through a two-proportion z-test using a difference in proportion analysis.

Survey Questions	African American (N=168)	Asian (N=24)	White (N=619)	P-Value
Question 1: How often do you have someone to take you to the doctor if you need it?				<0.001*
A little of the time	16 (9.5%)	4 (16.7%)	0 (0%)	
All of the time	62 (36.9%)	9 (37.5%)	619 (100%)	
Most of the time	40 (23.8%)	7 (29.2%)	0 (0%)	
None of the time	13 (7.7%)	0 (0%)	0 (0%)	
Some of the time	31 (18.5%)	4 (16.7%)	0 (0%)	
Missing	6 (3.6%)	0 (0%)	0 (0%)	
Question 2: How much do you agree or disagree that there is a lot of crime in your neighborhood?				0.001*
Agree	30 (17.9%)	3 (12.5%)	52 (8.4%)	
Disagree	129 (76.8%)	21 (87.5%)	553 (89.3%)	
Missing	9 (5.4%)	0 (0%)	14 (2.3%)	
Question 3: The crime rate in my neighborhood makes it unsafe to go on walks at night. Would you say that you...				<0.001*
Agree	59 (35.1%)	5 (20.8%)	100 (16.2%)	
Disagree	86 (51.2%)	19 (79.2%)	486 (78.5%)	
Do not Know	12 (7.1%)	0 (0%)	13 (2.1%)	
Missing	11 (6.5%)	0 (0%)	20 (3.2%)	
Question 4: How often are you treated with less courtesy than other people when you go to a doctor's office or other health care provider?				0.010*
Always	2 (1.2%)	0 (0%)	4 (0.6%)	
Most of the time	5 (3.0%)	0 (0%)	7 (1.1%)	
Never	58 (34.5%)	13 (54.2%)	324 (52.3%)	
Rarely	64 (38.1%)	8 (33.3%)	177 (28.6%)	
Sometimes	37 (22.0%)	3 (12.5%)	98 (15.8%)	
Missing	2 (1.2%)	0 (0%)	9 (1.5%)	
Question 5: How often are you treated with less respect than other people when you go to a doctor's office or other health care provider?				0.023*
Always	3 (1.8%)	0 (0%)	5 (0.8%)	
Most of the time	4 (2.4%)	0 (0%)	10 (1.6%)	
Never	54 (32.1%)	13 (54.2%)	312 (50.4%)	
Rarely	65 (38.7%)	7 (29.2%)	177 (28.6%)	
Sometimes	34 (20.2%)	4 (16.7%)	101 (16.3%)	
Missing	8 (4.8%)	0 (0%)	14 (2.3%)	
Question 6: How often do you feel like a doctor or nurse is not listening to what you were saying when you go to a doctor's office or other health care provider?				0.002*
Always	6 (3.6%)	0 (0%)	15 (2.4%)	
Most of the time	12 (7.1%)	0 (0%)	36 (5.8%)	
Never	68 (40.5%)	13 (54.2%)	163 (26.3%)	
Rarely	38 (22.6%)	7 (29.2%)	190 (30.7%)	
Sometimes	38 (22.6%)	4 (16.7%)	201 (32.5%)	
Missing	6 (3.6%)	0 (0%)	14 (2.3%)	
Missing	13 (7.7%)	0 (0%)	17 (2.7%)	
Question 7: How often do you receive poorer service than others when you go to a doctor's office or other health care provider?				<0.001*
Always	3 (1.8%)	0 (0%)	7 (1.1%)	
Most of the time	3 (1.8%)	1 (4.2%)	9 (1.5%)	
Never	57 (33.9%)	10 (41.7%)	330 (53.3%)	
Rarely	53 (31.5%)	12 (50.0%)	186 (30.0%)	
Sometimes	43 (25.6%)	1 (4.2%)	70 (11.3%)	
Missing	9 (5.4%)	0 (0%)	17 (2.7%)	
Question 8: Within the past 12 months, were you worried whether the food you had bought just did not last and you did not have money to get more?				<0.001*
Never true	117 (69.6%)	21 (87.5%)	548 (88.5%)	
Often true	11 (6.5%)	1 (4.2%)	20 (3.2%)	
Sometimes true	33 (19.6%)	2 (8.3%)	51 (8.2%)	
Missing	7 (4.2%)	0 (0%)	0 (0%)	
Missing	11 (6.5%)	0 (0%)	15 (2.4%)	
Question 9: It is within a 10–15-minute walk to a transit stop (such as bus, train, trolley, or tram) from my home. Would you say that you...				<0.001*
Agree	111 (66.1%)	18 (75.0%)	314 (50.7%)	
Disagree	42 (25.0%)	4 (16.7%)	262 (42.3%)	
Do not Know	4 (2.4%)	2 (8.3%)	28 (4.5%)	
Missing	11 (6.5%)	0 (0%)	15 (2.4%)	

Analysis of the social determinants of health survey responses yielded significant disparities across ethnicities for the same survey questions. Question 1 did not yield statistically significant differences (p=0.225) in access to transportation; however, significant disparities were noted in perceptions of neighborhood crime (p=0.003), and feelings of safety during nighttime walks (p=0.003) with higher levels of agreeance in Hispanics. Significant disparities were noted amongst the Hispanic responses regarding feeling heard by healthcare providers (p=0.009), however, no significant differences were noted in terms of receiving inadequate service, a lack of respect, or less courtesy. Significant differences were observed in worries about food security (p<0.001) and the proximity of transit stops (p=0.005) with Hispanic participants showing higher levels of concern and agreement, respectively (Table 2).

**Table 2:** Social Determinants of Health Survey questions and responses for Hispanic and non-Hispanic ethnicities. A difference of proportions analysis with p-values is indicated for each question. Data is presented throughout as 'n(%)' (*) represents significance in relation to p<0.05 Significance was determined through a two-proportion z-test using a difference in proportion analysis.

Survey Questions	Hispanic (N=122)	Non-Hispanic (N=810)	P-Value
Question 1: How often do you have someone to take you to the doctor if you need it?			0.225
A little of the time	16 (13.1%)	88 (10.9%)	
All of the time	41 (33.6%)	322 (39.8%)	
Most of the time	29 (23.8%)	218 (26.9%)	
None of the time	12 (9.8%)	44 (5.4%)	
Some of the time	22 (18.0%)	128 (15.8%)	
Missing	2 (1.6%)	10 (1.2%)	
Question 2: How much do you agree or disagree that there is a lot of crime in your neighborhood?			0.003*
Agree	23 (18.9%)	84 (10.4%)	
Disagree	92 (75.4%)	704 (86.9%)	
Missing	7 (5.7%)	22 (2.7%)	
Question 3: The crime rate in my neighborhood makes it unsafe to go on walks at night. Would you say that you...			0.003*
Agree	36 (29.5%)	163 (20.1%)	
Disagree	71 (58.2%)	592 (73.1%)	
Do not Know	8 (6.6%)	25 (3.1%)	
Missing	7 (5.7%)	30 (3.7%)	
Question 4: How often are you treated with less courtesy than other people when you go to a doctor's office or other health care provider?			0.555
Most of the time	2 (1.6%)	12 (1.5%)	
Never	66 (54.1%)	391 (48.3%)	
Rarely	34 (27.9%)	250 (30.9%)	
Sometimes	16 (13.1%)	139 (17.2%)	
Always	0 (0%)	6 (0.7%)	
Missing	4 (3.3%)	12 (1.5%)	
Question 5: How often are you treated with less respect than other people when you go to a doctor's office or other health care provider?			0.282
Most of the time	1 (0.8%)	14 (1.7%)	
Never	66 (54.1%)	375 (46.3%)	
Rarely	34 (27.9%)	250 (30.9%)	
Sometimes	15 (12.3%)	140 (17.3%)	
Always	0 (0%)	8 (1.0%)	
Missing	6 (4.9%)	23 (2.8%)	
Question 6: How often do you feel like a doctor or nurse is not listening to what you were saying when you go to a doctor’s office or other health care provider?			0.009*
Most of the time	14 (11.5%)	47 (5.8%)	
Never	45 (36.9%)	239 (29.5%)	
Rarely	30 (24.6%)	230 (28.4%)	
Sometimes	28 (23.0%)	253 (31.2%)	
Always	0 (0%)	21 (2.6%)	
Missing	5 (4.1%)	20 (2.5%)	
Question 7: How often do you receive poorer service than others when you go to a doctor's office or other health care provider?			0.516
Most of the time	3 (2.5%)	13 (1.6%)	
Never	64 (52.5%)	395 (48.8%)	
Rarely	32 (26.2%)	251 (31.0%)	
Sometimes	15 (12.3%)	114 (14.1%)	
Always	0 (0%)	10 (1.2%)	
Missing	8 (6.6%)	27 (3.3%)	
Question 8: Within the past 12 months, were you worried whether the food you had bought just did not last and you did not have money to get more?			< 0.001*
Never true	86 (70.5%)	687 (84.8%)	
Often true	7 (5.7%)	32 (4.0%)	
Sometimes true	27 (22.1%)	84 (10.4%)	
Missing	2 (1.6%)	7 (0.9%)	
Question 9: It is within a 10–15-minute walk to a transit stop (such as bus, train, trolley, or tram) from my home. Would you say that you...			0.005*
Agree	86 (70.5%)	444 (54.8%)	
Disagree	30 (24.6%)	308 (38.0%)	
Do not Know	3 (2.5%)	33 (4.1%)	
Missing	3 (2.5%)	25 (3.1%)	

## Discussion

The purpose of this study is to recognize the differences and inequalities seen in SLE patients of different racial and ethnic backgrounds. The main focus was on African Americans, Asians, whites, Hispanics, and non-Hispanic groups. This study is unique because it shows the interconnectedness of racial groups with different social determinants of health regarding the care of SLE patients. Social determinants of health are the conditions in which people live, work, play, and form connections that eventually affect their health and quality of life. While there are a multitude of different determinants, this study focuses on access to transportation, crime and safety, food security, and provider bias. The SDoH may be playing a larger role in the long-term management of SLE across race and ethnicity. Research has shown that factors including diet, housing stability, and education have an impact on systemic immune responses in autoimmune diseases, especially lupus [15]. Many studies concentrate on one factor individually, but this study evaluates four different variables and how each element affects the other regarding racial and ethnic groups, SDoH, and disease management. Not only does this article assess the interdependence of these elements, but it also highlights differences in SDoH for SLE disease management across various racial and ethnic groups. 

Access to transportation

The African American and Asian participants with lupus were less likely to have someone take them to the doctor compared to white individuals. However, on the other hand, African Americans and Asians with lupus have a transit stop from their home within a 10- to 15-minute walk compared to white individuals. Access to transportation is influenced by variables such as socioeconomic status, education, and geography. People living in urban areas are closer to public transportation, whereas people living in rural areas are not able to utilize public transit options [16]. Individuals with a lower socioeconomic status may not be able to afford a car, which further hinders their access to transportation. Those without a driver’s license or individuals who are unable to afford a car limit their access to transportation. Furthermore, education also plays a large role in income potential [16]. Those who have a lower education may be more reliant on public transit and carpooling, while those with a higher education have access to private vehicles for transportation [16]. Although our study does not directly assess income and education due to the lack of survey questions, these factors are important SDoHs that impact access to transportation. The lack of transportation has led to a series of adverse health effects, such as interrupted continuity of care, chronic illness complications, increased hospital readmissions, delays in the diagnosis of diseases, missed diagnostic testing, and medication noncompliance [17]. Many chronic autoimmune diseases, specifically SLE, have constantly changing guidelines and treatment options. There are newer medications on the rise with better efficacy for certain complications, along with more diagnostic tests for assessing the improvement of diseases. Many ethnic and racial groups are hindered from utilizing these new medications because of transportation barriers. Additionally, this study shows that African Americans, Asians, and Hispanics have a transit stop 10-15 minutes away from their homes, which could be explained by the areas they live in. Studies have shown that poor Hispanics, immigrants, and African Americans are more likely to live in bigger metropolitan areas with inconsistent public transportation options [18]. Furthermore, a higher percentage of African Americans and Hispanics do not have access to a vehicle compared to their white counterparts when trying to go to their doctors’ appointments [19]. When comparing private and public transportation, it is easier to reach the doctor’s office or pharmacy for medication refills when relying on your own vehicle. Amongst individuals with private transportation, they are more likely to have family or friends who are willing to give them a ride, lend them their car, or accompany them to their appointments [19]. On the other hand, individuals who utilize public transportation tend to worry about traffic congestion, time delays, complex transit routes, and multiple stops, which can lead to missed doctor’s appointments [20]. Since SLE is a chronic condition that is generally managed by the specialist care of a rheumatologist, it is important to consider the distance between traveling to a rheumatologist and traveling to their primary care doctor. There are far fewer rheumatologists than primary care physicians, forcing patients to travel further distances, burden correlates to additional poor health outcomes and increased disease severity for SLE patients who face obstacles with transportation [21], which can be especially difficult with public transportation. One study shows that 40% of lupus patients found it more difficult to travel to their rheumatologist, while only 20% found it difficult to travel to their primary care physician [21]. Approximately 90% of rheumatologists practice in a metropolitan area, 3% of rheumatologists practice in a micropolitan area, and 7% of rheumatologists practice in rural areas, creating a burden for individuals who live in rural areas with limited access to public transportation [22]. The extra burden correlates to additional poor health outcomes and increased disease severity for SLE patients who face obstacles with transportation [21]. 

Crime and safety

Individuals living in neighborhoods with increased crime are more likely to live in fear, causing constant anxiety. This can lead to an increased risk of consequential cardiovascular issues, lung disease, stroke, and arthritis [23]. Stress has a detrimental impact on the severity of their lupus symptoms and has been associated with increased flare-ups [24]. Stress may also lead to increased inflammation, triggering lupus flare-ups such as fatigue, joint pain, and body aches [24]. These effects may additionally impact reproductive health, depression, and anxiety amongst SLE patients in certain racial and ethnic groups [25]. Our results suggest that African Americans were more likely to agree that there are higher crime rates in their neighborhood, making them feel unsafe to walk at night in their neighborhood. Hispanic participants were also more likely to agree that there is more crime in their neighborhoods. There are many correlations between the socioeconomic status of a neighborhood and crime rates. Studies have shown that neighborhoods with increased poverty, including low-income and high unemployment rates, have higher crime rates than wealthier areas [26]. Being exposed to crime can negatively impact physical health, mental health, and social well-being [27]. Additional studies have shown that individuals who do not feel safe stepping outside their houses are more likely to have sedentary lifestyles and not get enough physical exercise [28]. As a result of this, there are higher rates of obesity and high blood pressure in African American and Hispanic groups, increasing the risk of SLE [29]. Crime has not only been a major stressor in these racial groups’ lives, but it has had negative health effects as well [27].

Food security**

The African American population, among other races, and the Hispanic population, among other ethnicities, were worried about their current food options and having enough money to buy more. Living in food-scarce areas has been linked to an increase in waist-to-height ratios among people [30]. Research has shown an increase in SLE incidence among obese individuals [29]. Additionally, obesity is a risk factor for worsening SLE symptoms and disease progression [31]. Research has found that a proper diet is an essential tool in the management of SLE symptoms. The immunomodulatory effects of low-calorie, low-protein foods with added fiber, polyunsaturated fatty acids, vitamins, minerals, and polyphenols can aid in maintaining homeostasis and increase periods of remission from flares [32,33]. 

Limited access to adequate food resources has been implicated as a risk factor for diabetes, hypertension, depression, and even psychological disorders [30]. In the United States, black and Hispanic-headed households have been found to experience a higher rate of food insecurity compared to other races and ethnicities [30]. Furthermore, food insecurity has been associated with obesity in women of all races [34]. This link to obesity may be due to the constant cycling between a state of hunger and periods of overindulgence in high-calorie, nutrient-poor, and low-cost foods. Binge eating has also been associated with individuals in food-insecure areas and can harm metabolic processes, shifting the body into a state of lipogenesis [30]. Furthermore, black and Hispanic households are more likely to be located in areas with limited supermarkets and grocery stores but with increased fast-food restaurants, convenience stores, and neighborhood stores [30].

Healthcare professional bias**

This study indicates that African Americans with SLE were more likely to experience provider dissatisfaction than the other races. In terms of SLE management and diagnosis, a study done in 2021 found that healthcare providers had significantly less confidence when assessing SLE-related rashes in patients with darker skin color compared to white patients [35]. The combination of healthcare provider attitudes affected by implicit bias along with a decreased ability to confidently diagnose certain conditions in people of color is likely a cause of increased disease severity and poor health outcomes among certain racial groups. This pattern of patient care is seen in several clinical settings and may have implications for the incidence and severity of SLE among black and Hispanic patients.

Furthermore, implicit bias is more likely to be implemented in stressful work conditions where patient overload is common [36]. Implicit biases are ingrained unconscious thoughts that may be difficult to consciously recognize and eliminate [37]. Many healthcare providers harbor implicit bias, which perpetuates negative attitudes toward black patients compared to positive attitudes toward their white counterparts [37]. These subtle implicit attitudes toward patients of color have been found to influence the quality of care and treatment choices. These provider attitudes lead to longer wait times for assessment and treatment, less time spent with patients, the use of a condescending tone in patient interactions, less thorough diagnostic work, and the assumption of treatment adherence capabilities [37]. Such detrimental attitudes toward patient care led to a decreased rate of patient satisfaction and trust in healthcare providers among targeted ethnic groups [37]. One such study found that among black college students in Georgia, implicit provider attitudes of stereotyping and lack of communication have led to a reluctance to seek future care, fostered distrust when treated by providers who are not from the same racial group, decreased adherence to treatment, and increased preference for home remedies [38]. Influenced beliefs like these may lead to poorer health outcomes in chronic conditions requiring regular management, like SLE. Another study comparing blood pressure control amongst non-Hispanic Black Americans and non-Hispanic White Americans indicated that the non-Hispanic Black Americans had higher rates of uncontrolled hypertension (34%) compared to their non-Hispanic White (28%), Asian (25%), and Hispanic (27%) counterparts. The 21% difference among non-Hispanic Black Americans accounted for 14% of missed visits with their physicians [39]. There are several hypotheses behind the missed visits, with provider bias being one of them [40]. As implicit bias plays a role in the long-term management of conditions such as hypertension, considerations taken to address this bias are crucial among patients with SLE, as chronic autoimmune diseases require careful and continuous monitoring to prevent the progression of the disease. 

Limitations and future directions

Our study has certain limitations. Data regarding social determinants like access to transportation, crime and safety, food security, and provider bias were collected via surveys and self-reported data. There is a possibility of response bias from the health survey data, affecting the validity of the results. The number of responses to the optional survey may alter the prevalence of SLE amongst the chosen population. Another limitation is the smaller sample size for the different population groups. There was a limited number of Asian participants compared to African American and white participants, which could alter the external validity of the Asian American lupus patients’ perspective on social determinants of health. An additional limitation of this manuscript may be that while it focuses on the American population, it may be challenging to draw specific conclusions about African American and Asian individuals within this population. However, the data from the All of Us database provides a more representative sample of the diversity within the United States. This database can thus offer a more comprehensive understanding of these groups within the broader American context.

Addressing social determinants that affect the quality of healthcare among various racial and ethnic groups is a complex undertaking. Healthcare providers should take steps to implement self-awareness and mindfulness to better combat the effects of implicit bias. Educational tools used in the training of medical professionals should be more inclusive of various skin colors to decrease the chances of missed diagnoses and low medical provider confidence in the clinical setting. Furthermore, community programs to decrease transportation issues, food scarcity, and neighborhood safety would be beneficial in addressing barriers to care that many African American and Hispanic patients encounter in daily life. It would be beneficial to expand public transit options in metropolitan and non-metropolitan areas to accommodate all individuals. Having reliable transportation can lead to a significant decrease in “no-show” doctors’ appointments. Increased utilization of telemedicine and home visits would be a beneficial tool to ensure those with less limited access to transportation could receive medical care. It is vital to reduce crime rates in neighborhoods and create a safer community. This can be done by improving connectivity in the streets, decreasing easy access to alcohol, and improving community infrastructure. 

## Conclusions

This study investigated the effect of unaddressed social health determinants in the management of SLE among various racial and ethnic groups; primarily researched were access to transportation, crime and safety, food security, and provider bias. Regarding transportation, African Americans and Asians with SLE are less likely to have someone take them to the doctor and believe they have higher rates of neighborhood crime, making them feel unsafe to go on walks. These racial groups and Hispanic participants had more concerns regarding food security. African American participants with SLE experienced being treated with less courtesy and respect during doctors’ visits, and Hispanic patients additionally felt as if they were not being heard by their doctors. As chronic autoimmune conditions are heavily influenced by social determinants of health due to their impact on systemic immune responses, management of these disparities amongst patients with SLE is critical. This study provides unique insight into possible reasons for the increased incidence of SLE among African Americans and Hispanic populations. The implications of this study incite a need for the implementation of policies to breach the barriers of care contributing to poor health outcomes among various racial and ethnic groups with SLE.
